# Cognitive development at late infancy and school age in children cooled for neonatal encephalopathy

**DOI:** 10.1038/s41390-025-04152-4

**Published:** 2025-05-30

**Authors:** Sara Rapuc, Sally Jary, Ross E. Vanderwert, David Odd, Ela Chakkarapani

**Affiliations:** 1 Population Health Sciences, Bristol Medical School, University of Bristol, Bristol, UK; 2 Translational Health Sciences, Bristol Medical School, University of Bristol, Bristol, UK; 3 Cardiff University Centre for Human Developmental Science, School of Psychology, Cardiff University, Cardiff, UK; 4 School of Psychology, Cardiff University, Cardiff, UK; 5 Division of Population Medicine, School of Medicine, Cardiff University, Cardiff, UK; 6 Neonatal Intensive Care Unit, St Michael’s Hospital, University Hospitals Bristol and Weston NHS Foundation Trust, Bristol, UK

## Abstract

**Background:**

We investigated the association and individual changes in cognitive scores between late infancy and early school age in children cooled for neonatal encephalopathy secondary to perinatal asphyxia (NE) who did not develop cerebral palsy.

**Methods:**

We included 50 children born ≥35 weeks gestation cooled for NE who did not develop cerebral palsy. We assessed cognition using an average of cognitive and language composite scores (CLC) from Bayley Scales of Infant and Toddler Development (Bayley-III) at 18–21 months and full-scale IQ (FSIQ) on Wechsler Intelligence Scale for Children (WISC-IV) at 6–8 years. Linear regression was used to assess the association between CLC and FSIQ.

**Results:**

Our cohort’s mean gestation was 39.8 (SD 1.6) weeks; 59% male. 80% had moderate NE. CLC scores were significantly associated with FSIQ (Coef 0.45 (95% CI: 0.17, 0.72), *R*^2^ 19%). About 45% of children’s cognitive scores lowered from 18 to 21 months to 6–8 years of age, with two FSIQ clusters differing by deprivation (7.3 vs 5.5, *p* = 0.009). Increasing CLC threshold to 95 still did not identify 63% having an FSIQ < 85.

**Conclusion:**

Bayley-III underestimates the delay at school age in children cooled for NE. Childhood IQ after NE appeared to be patterned by local deprivation.

**Impact:**

- Bayley-III underestimates school-age delays in children cooled for neonatal encephalopathy secondary to perinatal asphyxia (NE).
- Around 45% of children’s cognitive scores moved to a lower developmental range at school age.
- Increasing the Bayley-III threshold to 95 failed to identify nearly two-thirds of children with IQ < 85.
- Childhood IQ after NE appeared to be patterned by local deprivation.
- Longitudinal monitoring of children cooled for neonatal encephalopathy secondary to perinatal asphyxia is needed to support their cognitive development.

## Introduction

Therapeutic hypothermia (TH; or cooling therapy) improves survival and neurodevelopmental outcomes in children with neonatal encephalopathy secondary to perinatal asphyxia (NE) and is the standard care in high income countries.^1^ Due to TH, more children survive, and fewer survivors develop severe motor abnormalities like cerebral palsy (CP).^2,3^ However, children cooled for NE are still at high risk for cognitive deficits at early school age, even in the absence of CP.^4^ Therefore, it is important to identify those children at risk of cognitive difficulties at school age early on to offer timely and targeted support.

Routine follow-up of children cooled for moderate to severe NE at 12–24 months focuses on the assessment of cognitive and language development.^5^ In clinical practice, early assessment of development is often done using the Bayley Scales of Infant and Toddler Development to assess cognitive, language, and motor development, and allowing for separate composite scores for these areas to be derived at one-month age intervals.^6^ At early school age, cognitive abilities are typically assessed using the Wechsler Intelligence Scale for Children.^7^ It assesses multiple cognitive abilities that estimate a total intelligence quotient (full-scale IQ, FSIQ).^7^

Some previous studies conducted in children with, and without risk, for cognitive deficits found weak to good associations between Bayley Scales of Infant and Toddler Development, Third Edition (Bayley-III) cognitive scores and Wechsler Intelligence Scale for Children, Fourth Edition (WISC-IV) FSIQ (*r* between 0.33–0.62).^8–11^ The associations tend to be stronger in children with higher risk of disability, as exemplified by a Swedish study where Bayley-III cognitive composite scores at 2.5 years explained up to 38% of the variance in WISC-IV FSIQ at 6.5 years in extremely preterm born children,^11^ but only 24% in full term born controls (multivariable model).^10^

While there is some evidence for an association between Bayley and WISC in the NE population from a randomized controlled trial of TH, this work used an earlier version of Bayley (version II), and the results appeared less predictive for scores between 70 and 84.^12^ The study found that Bayley-II Mental Developmental Index (MDI) < 70 had a high predictive value for FSIQ < 70 at school age (sensitivity = 0.87, specificity = 0.96).^12^ However, this association was not robust when Bayley-II MDI was between 70 and 84; only half of the children with moderate to severe NE and without CP (13/27) remained in the aforementioned developmental category at 6–7 years of age.^12^ In contrast, most of the children with CP remained in the same developmental category, but that might be due to the majority (96%) having severe cognitive impairments (MDI and FSIQ < 70).^12^

Data on the association between cognition assessed using Bayley-III, which is commonly used in clinical practice in the therapeutic hypothermia era, and cognition at school age in children without severe motor disabilities after NE is unknown. Further, the evolution of cognitive scores in this population from 18 months to early school age is not well described. We hypothesized that the Bayley-III CLC scores at 18–21 months will be positively associated with cognitive scores at 6–8 years. The aim of this study was to evaluate the association and individual changes in cognitive scores assessed using Bayley-III average cognitive and language composite (CLC) scores at 18–21 months and WISC-IV FSIQ at 6–8 years in children cooled for moderate to severe neonatal NE.

## Methods

### Study design

Data was drawn from the CoolMRI study,^13^ which recruited from a population-based cohort of children aged 6–8 years without cerebral palsy who had received TH for moderate to severe NE after birth. The eligible children were identified through the patient database at St Michael’s Hospital, Bristol, UK. The children had been born at or above 35 weeks gestation between April 2008 and December 2012 in South West England and underwent cooling therapy at St Michael’s Hospital, Bristol. All children had evidence of perinatal asphyxia and moderate-severe encephalopathy confirmed by clinical neurological examination and amplitude-integrated EEG.^14^ Absence of CP at 18–21 months and at 6–8 years of age, was confirmed following detailed neurological examination by an experienced pediatrician.^15^ Children were followed up at 18–21 months and 6–8 years of age. Developmental assessments were conducted by an experienced physiotherapist (SJ) at 18–21 months, and an experienced neuropsychologist at 6–8 years of age.^4^ Assessors were blinded to the clinical details of the children, although at 18–21 months the assessor was aware that children underwent TH.

### Cognitive assessments

Recruited children were followed longitudinally, with assessment at two time points. At 18–21 months, children were assessed with the Bayley-III.^6^ Bayley-III Cognitive and Language Composite (CLC; average of the Bayley-III Cognitive Composite and Bayley-III Language Composite scores) score was used in the analysis as language represents an essential part of cognitive assessment.^16^ Cognitive abilities at 6–8 years of age were assessed with the WISC-IV.^7^ Full-Scale IQ was used in the analysis. Both assessments have a standardized mean of 100 and a standard deviation (SD) of 15.

### Data collection

Baseline data collected by researchers in the CoolMRI study included gestation at birth, birth weight, measures of asphyxia, and encephalopathy. The severity of asphyxia was defined by acidosis on the blood gas obtained within 1 h after birth, Apgar score, and need for respiratory support at 10 min after birth.^14^ We used the severity of abnormalities on neurological examination to denote the severity of NE (mild, moderate, or severe). The severity of encephalopathy was also evaluated with amplitude-integrated EEG, acquired prior to cooling therapy, and classified as moderately or severely abnormal by an experienced neonatologist. The total neonatal brain MRI injury score was calculated based on a qualitative assessment of structural neonatal brain T1-weighted MRI scans at 4–15 days post-birth by an experienced perinatal neurologist,^15^ using the Rutherford classification system.^17^ A total neonatal brain MRI injury score was used in the analysis (range: 0–11; 11 represents maximum injury) and computed as a sum of injury across basal ganglia and thalami, cortex, white matter, and posterior limb of internal capsule.^17^ The socio-economic status was estimated by the index of multiple deprivation (IMD).^13^ IMD is a measure of relative deprivation in small areas in England and is computed as a weighted sum of (1) income deprivation, (2) employment deprivation, (3) education, skills and training deprivation, (4) health deprivation and disability, (5) crime, (6) barriers to housing and services, and (7) living environment deprivation.^18^ IMD was calculated from the 2019 release for England and reported as deciles, ranging from 1 to 10. A score of 1 represents the most deprived 10% of small areas in England, and a score of 10 represents the least deprived 10% of small areas in England.^13^ The IMD deciles were calculated from the mother’s postcode at 6–8 years.

### Sample size and outcomes

The CoolMRI study identified a total of 69 eligible children through the St Michael’s Hospital patient database, of whom 50 were recruited in the study.^15^ Primary outcome measure was the FSIQ at 6–8 years of age.

### Statistical analysis

Statistical analyses were conducted using R.^19^ Complete case analyses were used for all statistical analyses. The evaluation of whether the cognitive scores are normally distributed was carried out by quantile–quantile (Q–Q) plots and the Shapiro-Wilk normality test. Density plots were further used to visualize the distribution of cognitive scores. The main analysis tested for association between Bayley-III CLC and WISC-IV FSIQ scores. The means of the Bayley-III CLC and WISC-IV FSIQ were compared with a paired t-test. Cognitive outcomes at 18–21 months and 6–8 years were categorized using the following cut-off points: < 70, 70–84, 85–100, and > 100. The percentage of children with scores remaining in the same category, moving to a lower or higher developmental category between the two assessments was calculated. Univariable and multivariable linear regression models were used to predict WISC-IV FSIQ scores. In the first model, only Bayley-III CLC was used as a predictor. In the second model, the contributions of age at Bayley-III assessment, sex, birth weight, gestation at birth, and IMD were evaluated. In the third and fourth models, the contributions of neonatal brain MRI injury score and NE clinical grade were evaluated. The Akaike Information Criterion (AIC) was used to compare the four models. No external validation data were available. In a further sub-group data analysis, the characteristics of children who deteriorated and children who remained in the same range or progressed to a higher FSIQ score at 6–8 years of age, were compared using the independent sample t-test for continuous and the Pearson’s *χ*^2^ with Yates’ continuity correction for categorical variables. The median test (available in R package agricolae) was used to compare the medians of IMD and neonatal brain MRI injury scores between the groups. In a further sensitivity analysis, the regression analyses were repeated using IMD as a binary predictor (IMD lower band: < 6 vs IMD upper band: 6–10).

## Results

### Participant characteristics

Of the 69 invited children, 19 were excluded at 6–8 years (11 did not respond, 5 moved away, and 3 declined participation).^15^ Of the remaining 50 recruited children, a total of 49 (98%) children had available data for both Bayley-III CLC and WISC-IV FSIQ. These were used in the main analysis. The sociodemographic information (including IMD), clinical characteristics, neonatal brain MRI injury score, and Bayley-III cognitive and language composite scores of children who were eligible but not recruited in the study were not significantly different to those included in this study (Table S1).

Baseline characteristics of children included in the main analysis are presented in Table 1. The mean gestational age of the group was 39.8 (SD 1.6) weeks, and over half (*n* = 29, 59%) were male. Most children (*n* = 39, 80%) were classified at birth with a moderate NE grade, while 10 (20%) with severe NE grade. At 6–8 years, the median IMD was 7 (IQR 4–9). There was no significant association between IMD decile and NE grade (*χ*²(9) = 8.35, *p* = 0.499).

**Table 1:** Baseline characteristics of the study cohort.

Characteristics	Participants (*n* = 49)
Neonatal	
Sex, no. *(%)* male	29 (59)
Birth weight (g), *mean (SD)*	3376 (519)
Gestational age (weeks), *mean (SD)*	39.8 (1.6)
Worst pH within 1 h of life, *mean (SD)*	6.9 (0.2)
Worst base excess within 1 h of life, *mean (SD)*	−17.8 (7.0)
Apgar score at 10 min, *median (IQR)*	6 (5–8)
Ventilation at 10 min, no. *(%)*	34 (69)
aEEG category	
Moderately abnormal, no. (*%*)	46 (94)
Severely abnormal, no. (*%*)	3 (6)
NE grade	
Moderate, no. *(%)*	39 (80)
Severe, no. *(%)*	10 (20)
Age at neonatal MRI (days), *median (IQR)*	8 (7–9)
Neonatal MRI brain injury score, *median (IQR)*	2 (0–3)
6–8 years	
Weight (kg), *mean (SD)*	25.3 (4.8)
Head circumference (cm), *mean (SD)*	52.4 (2.0)
Index of multiple deprivation, *median (IQR)*	7 (4–9)

The data presented are for the participants included in the main analysis.

*NE* neonatal encephalopathy secondary to perinatal asphyxia.

### Bayley-III CLC & WISC-IV FSIQ scores

**Fig. 1:**
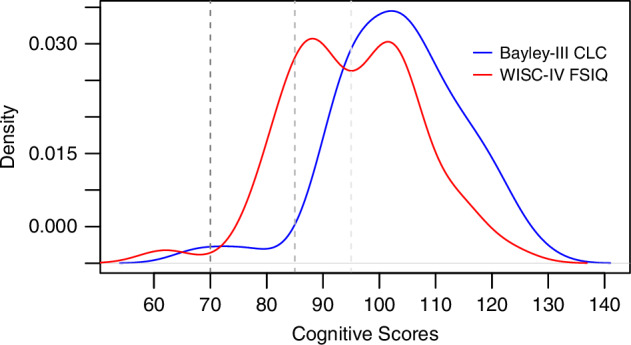
Density plots of Bayley-III Cognitive & Language Composite scores at 18–21 months and WISC-IV Full-Scale IQ at 6–8 years.

The distribution of scores at both time points is presented in Fig. 1. There was little evidence that the scores were not normally distributed (*p* = 0.193 and *p* = 0.389), although the FSIQ density plot visually suggested two distinct peaks/FSIQ clusters (Fig. 1). Therefore, a post-hoc K-means cluster analysis of FSIQ (cluster number = 2) was conducted and two clusters were compared by sex, NE clinical grade, neonatal brain MRI injury score, and IMD, using independent sample t-test for continuous and χ^2^ tests for categorical variables. The FSIQ clusters significantly differed by IMD, *t*(43.76) = 2.72, *p* = 0.009. The mean IMD for Cluster 1 (mean FSIQ 104.8, SD 6.6) was 7.3 (less deprived areas), while for Cluster 2 (mean FSIQ 85.6, SD 6.5) was 5.5 (more deprived areas). No statistically significant differences were found in sex, NE clinical grade, and neonatal brain MRI injury score.

Cognitive scores at 18–21 months and 6–8 years are reported in Table 2. The Bayley-III CLC scores (mean 103.4, SD 11.3) were significantly higher than WISC-IV FSIQ scores (mean 95.4, SD 11.7), *t*(48) = 4.61, *p* < 0.001. Most children had a Bayley-III CLC of 85–100 (*n* = 16, 32.7%) or > 100 (*n* = 31, 63.3%) at 18–21 months of age, with two (4.0%) children having a score of < 85. At 6–8 years of age, 23 (46.9%) and 18 (36.7%) children had a WISC-IV FSIQ of 85–100 or > 100, respectively, and 8 (16.3%) children had a WISC-IV FSIQ of < 85.

**Table 2:** Cognitive scores at 18–21 months and 6–8 years.

Cognitive outcomes	Participants (*n* = 49)
Bayley-III	
Age at assessment (months), *mean* (SD)	18.5 (0.6)
Cognitive composite, *mean* (SD)	103.7 (11.6)
Language composite, *mean* (SD)	103.2 (13.7)
Cognitive and language composite, *mean* (SD)	103.4 (11.3)
WISC-IV	
Age at assessment (years), *mean* (SD)	7.0 (0.5)
Full-scale IQ, *mean* (SD)	95.4 (11.7)
Full-scale IQ < 85, no. *(%)*	8 (16.3)
Verbal comprehension index, *mean* (SD)	97.6 (11.3)
Perceptual reasoning index, *mean* (SD)	92.9 (12.3)
Working memory index, *mean* (SD)	95.4 (13.1)
Processing speed index^a^, *mean* (SD)	99.9 (14.6)

Data presented are for the participants included in the main analysis.

^a^The variable had one missing value.

### Associations between scores

**Fig. 2:**
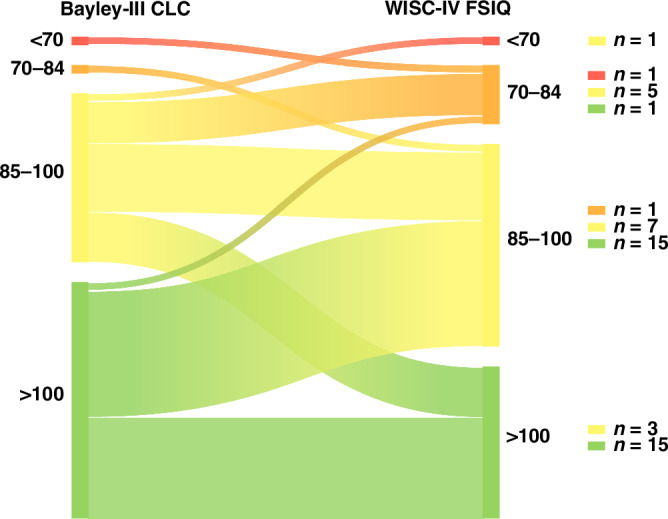
Individual changes in Bayley-III CLC scores at 18–21 months to WISC-IV FSIQ scores at 6–8 years of age. *Note*. CLC average of Cognitive & Language Composite scores, FSIQ Full-Scale IQ.

Individual changes in Bayley-III CLC and WISC-IV FSIQ scores over time are presented in Fig. 2. Most children’s scores either remained in the same developmental category (*n* = 22, 44.9%) or changed to a lower developmental category (*n* = 22, 44.9%). Only 5 (10.2%) of children’s scores moved to a higher developmental category at early school age. Children with a Bayley-III CLC between 85 and 100 (*n* = 16) had a range of WISC-IV FSIQ with 6 (37.4%) below 85, 7 (43.8%) between 85 and 100 and 3 (18.8%) above 100. Children with a Bayley-III CLC above 100 (*n* = 31) had a range of WISC-IV FSIQ with one (3.2%) below 85, 15 (48.4%) between 85–100 and 15 (48.4%) above 100. Of the remaining two children, one moved from a Bayley-III CLC of < 70 to a WISC-IV FSIQ of 70–84, and the other from a Bayley-III CLC of 70–84 to a WISC-IV FSIQ of 85–100.

### Regression analyses

**Fig. 3:**
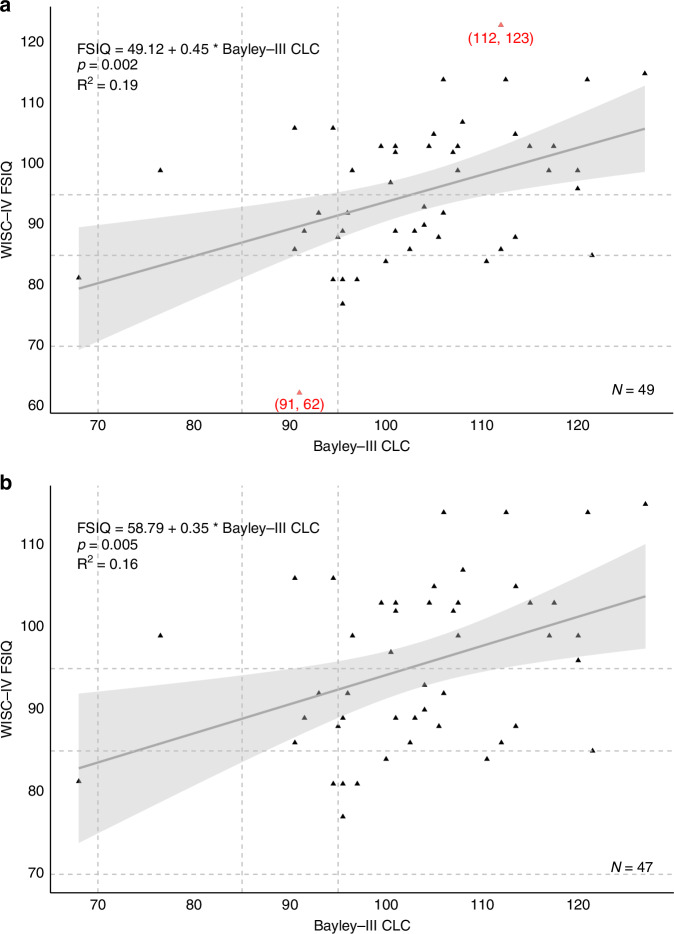
Linear relationship between Bayley-III CLC and WISC-IV FSIQ. Scatterplots show the association between Bayley-III CLC scores and WISC-IV FSIQ. **a** includes all data points, with outliers (absolute residuals > 20 points from zero) shown in red. **b** excludes these outliers. CLC average of Cognitive & Language Composite scores, FSIQ Full-Scale IQ.

The linear association between Bayley-III CLC and WISC-IV FSIQ is presented in Fig. 3 (unadjusted model). Reference lines indicate cut-off thresholds at 70, 85, and 95 for both assessments. Five out of 8 (62.5%) children with an WISC-IV FSIQ below 85 had a Bayley-III CLC above 95.

The results of all adjusted linear regression models are presented in Table 3. Bayley-III CLC scores were the only parameters that were significantly associated with WISC-IV FSIQ when adjusting for relevant sociodemographic factors, neonatal brain MRI injury score and NE clinical severity grade. When adding these parameters, the predictive ability of the models increased from 19% to 36% for FSIQ.

**Table 3:** Regression models of WISC-IV FSIQ at 6–8 years by Bayley-III CLC at 18–21 months.

	Regression coefficients			Model fit	
	Estimate	95% CI	*p*-value	*R*^2^	AIC
	Univariable model				
(Intercept)	49.12				
Bayley-III CLC	**0.45**	**0.17, 0.72**	**0.002**	0.19	374.58
	Multivariable models				
Model 1				0.26	380.36
(Intercept)	39.58				
Bayley-III CLC	**0.49**	**0.18, 0.81**	**0.003**		
Age at Bayley-III	−0.85	−6.41, 4.71	0.759		
Male	3.27	−3.73, 10.27	0.351		
Birth weight	0.00	−0.01, 0.00	0.554		
Gestational age	0.52	−2.05, 3.09	0.683		
IMD	0.79	−0.53, 2.10	0.233		
Model 2				0.32	378.03
(Intercept)	46.71				
Bayley-III CLC	**0.51**	**0.21, 0.81**	**0.002**		
Age at Bayley-III	−1.19	−6.59, 4.21	0.658		
Male	4.45	−2.44, 11.35	0.199		
Birth weight	0.00	−0.01, 0.00	0.406		
Gestational age	0.64	−1.85, 3.13	0.606		
IMD	0.55	−0.75, 1.84	0.400		
MRI injury score	−1.66	−3.39, 0.06	0.058		
Model 3				0.36	376.60
(Intercept)	66.47				
Bayley-III CLC	**0.49**	**0.19, 0.78**	**0.002**		
Age at Bayley-III	−1.38	−6.66, 3.91	0.601		
Male	2.96	−4.01, 9.93	0.396		
Birth weight	0.00	−0.01, 0.00	0.370		
Gestational age	0.31	−2.16, 2.78	0.803		
IMD	0.58	−0.69, 1.85	0.361		
MRI injury score	−0.48	−2.67, 1.72	0.664		
NE grade (severe:moderate)	−8.35	−18.26, 1.55	0.096		

The regression coefficients were adjusted for: (1) sociodemographic data [Model 1]; (2) variables in Model 1 and neonatal brain MRI injury score [Model 2]; and (3) variables in Model 2 and NE grade [Model 3].

*NE* neonatal encephalopathy secondary to perinatal asphyxia, *IMD* Index of multiple deprivation.

Changed estimates, 95% CIs and p-values of significant results to bold.

### Sub-group analysis

In a further sub-group analysis, we explored differences in characteristics of children whose scores changed to a lower developmental category (*n* = 22, 44.9%) compared to children who remained in the same or moved to a higher developmental category at 6–8 years of age (*n* = 27, 55.1%). Those children whose developmental category reduced between the two time points had significantly lower WISC-IV FSIQ scores than those with scores that remained in the same or moved to a higher developmental category. Addition, male infants were more likely than female infants to increase their scores over the time period (*p* = 0.040). There were no other significant differences between the two groups regarding clinical characteristics, neonatal brain MRI injury score, IMD, and Bayley-III CLC scores (Table 4).

**Table 4:** Comparison of baseline characteristics of children with unchanged or increased scores compared to children with scores that decreased between 18–24 months and 6–8 years.

Characteristics	Children with unchanged/increased scores (*n* = 27)	Children with decreased scores (*n* = 22)	*p*-value
Neonatal			
Sex			**0.040**
Female, no. *(%)*	**7 (26)**	**13 (59)**	
Male, no. *(%)*	**20 (74)**	**9 (41)**	
Birth weight (g), *mean (SD)*	3316 (490)	3449 (556)	0.382
Gestational age in weeks, *mean (SD)*	39.9 (1.4)	39.6 (1.8)	0.538
Worst pH, *mean (SD)*	7.0 (0.2)	6.9 (0.1)	0.326
Worst base excess, *mean (SD)*	−16.4 (6.2)	−19.4 (7.8)	0.141
Apgar score at 10 min, *median (IQR)*	6 (5–8)	6 (2–8)	0.740
Ventilation at 10 min, no. *(%)*	18 (67)	16 (73)	0.884
aEEG category			
Moderately abnormal, no. (*%*)	27 (100)	19 (86)	0.167
Severely abnormal, no. (*%*)	0 (0)	3 (14)	
NE grade			
Moderate, no. *(%)*	24 (89)	15 (68)	0.152
Severe, no. *(%)*	3 (11)	7 (32)	
Neonatal MRI brain injury score, *median (IQR)*	2 (0–3)	2.5 (1–4)	0.145
18–21 months			
Bayley-III CLC, *mean (SD)*	101.7 (12.9)	105.5 (8.8)	0.222
6–8 years			
Weight, *mean (SD)*	25.0 (4.2)	25.7 (5.5)	0.612
Head circumference, *mean (SD)*	52.3 (1.3)	52.5 (2.7)	0.682
WISC-IV FSIQ, *mean (SD)*	**101.7 (9.9)**	**87.5 (8.6)**	**<0.001**
Index of multiple deprivation, *median (IQR)*	7 (5–9)	6.5 (4–8)	0.367

*NE* neonatal encephalopathy secondary to perinatal asphyxia, *MRI* magnetic resonance imaging, *Bayley-III CLC* Bayley Scales of Infant and Toddler Development, Third Edition, average of Cognitive & Language Composite Score, *WISC-IV* *FSIQ* Wechsler Intelligence Scale for Children, Fourth Edition, Full-Scale IQ.

*p* values that are statistically significant are shown in bold.

### Sensitivity analysis

In a post-hoc analysis, when using IMD as a binary variable (defined as deciles 1–5 or 6–10) we saw similar results to the main analysis with Bayley-III CLC remaining the sole predictor of later FSIQ (Table S2).

## Discussion

We explored the associations between cognitive and language development assessed using Bayley-III at 18–21 months and WISC-IV at 6–8 years of age in children cooled for moderate to severe NE. While we found positive associations between Bayley-III CLC and WISC-IV FSIQ, the Bayley-III explained only a small proportion of variance in FSIQ at school age; with limited predictive value at 6–8 years for cognitive outcomes in our cohort of children with NE without CP.

Our findings are remarkably similar to other published research predicting childhood FSIQ from infant Bayley scores. In our sample, over a third of children with a Bayley-III CLC score of 85–100 had FSIQ below 85 at early school age, similar to a study in children cooled for moderate to severe encephalopathy without CP, where 11 of 33 with Bayley-II MDI above 84 at 18–22 had IQ below 84 at 6–7 years.^12^ In our study, Bayley-III CLC alone explained only 19% of the variance in FSIQ, increasing to 36% when sociodemographic factors (including measure of relative deprivation, IMD), neonatal brain MRI and NE severity, were included; however, the Bayley-III CLC alone better predicted FSIQ as shown by lower AIC. This is comparable to findings in preterm populations.^20^ Yet over 60% of FSIQ variance remains unexplained when using Bayley scores as a sole predictor.

Our study’s unique contribution to the work of other researchers on the limited association between Bayley and WISC, is that we also investigated individual cognitive trajectories and associated factors. Around 45% of children moved to a lower developmental category, whereas others stayed in the same developmental category or progressed to a higher one at school age. While sex was not a significant predictor of FSIQ, more male infants appeared to increase their score between the two time periods, and further work may be needed to identify the mechanisms behind this. No significant differences were found between children whose developmental category changed between the two time points in basic clinical characteristics, neonatal brain MRI injury score and IMD. Other factors might be driving these changes, such as brain plasticity or other protective mechanisms. Previous work has demonstrated significant positive associations between early environment/life experiences and later brain and cognitive development.^21,22^

The limited association between Bayley-III and WISC-IV may be explained by multiple factors.^10,11,23^ Bayley-III is used to assess children’s abilities at different points in development,^23^ while individual IQ scores on intelligence tests like WISC-IV tend to be relatively stable from childhood onwards and predictive of later cognitive performance.^24^ The limited association between the two measures may also be due to the lengthy interval between assessments, coupled with potential measurement errors. Yu and colleagues^25^ found that accounting for measurement errors and shorter intervals showed high stability in intelligence from early infancy through adulthood, challenging previous research. Furthermore, the complexity of skills measured by IQ tests may not be evident at earlier ages since cognitive abilities are still developing. Individual score changes over time may also be patterned by various environmental factors influencing individual cognitive trajectories. For instance, we found that children from less deprived areas had higher FSIQ scores than those from more deprived neighborhoods.

Bayley-III tends to underestimate developmental delay as compared to its previous version (version II) when using a standard cut-off of < 70 for moderate-severe delay.^26^ Other researchers found that by using higher thresholds (e.g., 95) on Bayley-III cognitive composite scores, the sensitivity to detect developmental delay increased.^9,11^ In our study, increasing the Bayley-III threshold to 95 did not improve identification of children with a WISC-IV FSIQ < 85, as 62.5% of children still had FSIQ below 85. In 2019, Bayley-IV was published and is being integrated into clinical practice.^27^ Bayley-IV did not include high-risk populations in the standardization sample. Bayley-III included 10% of children at risk for developmental delay which is thought to be the reason for inflated scores.^26,27^ The sensitivity of Bayley-IV to detect later developmental delay should be evaluated in future studies.

The results of this study need to be assessed in line with the following limitations. Due to the small sample size, we were limited to performing only simple analyses and the results should be interpreted with caution. Furthermore, the Bayley-III was administered at an early age (at 18–21 months), whereas stronger associations between Bayley-III and WISC-V were shown when Bayley-III was administered at 31–42 (*r* = 0.474) months than at 6–18 months (*r* = 0.157).^28^ Additionally, we did not have a Bayley-III social-emotional scale to be compared to FSIQ at 6–8 years.

Nevertheless, our study’s strengths include its longitudinal design and focus on individual cognitive trajectories, an aspect often overlooked in the studies. A further strength is the inclusion of children without CP who constitute the majority (85–88%) of survivors following TH for NE.^29^ This is unique as studies often include children with severe developmental problems where predictions of outcomes are easier. Furthermore, most children in our sample had moderate encephalopathy, and only a few had severe developmental delay in FSIQ at school age. Our study suggests that there might be unique challenges in finding associations/predictions in children with subtle problems and moderate delay.

In conclusion, our findings suggest limitations in using Bayley-III scores before 2 years as a prognostic tool for the identification of children at risk for cognitive impairments at school age. Clinicians should be therefore aware of the limited predictive ability of Bayley at an early age and clinical practice should be adapted to include longitudinal following of children at risk into early school age. Longitudinal monitoring, which is not currently part of routine clinical practice, might be beneficial for accurate identification and support of children’s cognitive development.

## Supplementary information

Supplementary information

## Data Availability

All data generated or analyzed during this study are included in this published article.
