# Supplementary material for: Cognitive development at late infancy and school age in children cooled for neonatal encephalopathy
Source: Pediatr Res. 2025 May 30;99(1):315–22. doi: 10.1038/s41390-025-04152-4 (PMC12920137; doi:10.1038/s41390-025-04152-4)
Supplement: Supplementary file 1 — Supplementary information [file 41390_2025_4152_MOESM1_ESM.pdf]

Table S1. Comparison of characteristics of children who took part in the study and eligible children who were not recruited in the CoolMRI study.

Characteristics	Children who took part in the study (n = 49)	Not recruited children (n = 19)	*p*-value
Sex, no. *(%)* male	29 (59)	11 (58)	1.0
Birth weight (g), *mean (SD)*	3376 (519)	3,526 (778)[a]	.506
Gestational age (weeks), *mean (SD)*	39.8 (1.6)	39.2 (1.2)[a]	.127
Worst pH within 1 h of life, *mean (SD)*	6.9 (0.2)	7.0 (0.2)[a]	.095
Apgar score at 10 min, *median (IQR)*	6 (5-8)	6 (4-9)[b]	.538
aEEG category			
Moderately abnormal, no. (*%*)	46 (94)	13 (87)	.718
Severely abnormal, no. (*%*)	3 (6)	2 (13)	
Neonatal MRI brain injury score, *median (IQR)*	2 (0-3)	1 (1-3)[b]	.556
Index of Multiple Deprivation, *median (IQR)*	7 (4-9)	5.0 (1.5-7.5)[c]	.145
Cognitive composite, *mean* (*SD*)	103.7 (11.6)	96.9 (16.8)[b]	.192
Language composite, *mean* (*SD*)	103.2 (13.7)	93.3 (15.3)[b]	.050
Cognitive and language composite, *mean* (*SD*)	103.4 (11.3)	95.1 (15.1)[b]	.083

[a]5 missing values, [b]6 missing values, [c]7 missing values.

Table S2. Regression models of WISC-IV FSIQ at 6-8 years by Bayley-III CLC at 18-21 months using IMD as binary predictor.

	Regression coefficients			Model fit	
	Estimate	95% CI	*p*-value	R^2	AIC
	**Univariable model**				
(Intercept)	49.12				
Bayley-III CLC	0.45	0.17, 0.72	**.002**	0.19	374.58
	**Multivariable models**				
Model 1				0.25	380.52
(Intercept)	40.66				
Bayley-III CLC	0.49	0.17, 0.81	**.003**		
Age at Bayley-III	-0.91	-6.48, 4.65	.742		
Male	3.74	-3.08, 10.56	.275		
Birth weight	0.00	-0.01, 0.00	.457		
Gestational age	0.64	-1.92, 3.19	.617		
IMD (upper:lower band)	4.00	-3.02, 11.01	.257		
Model 2				0.31	378.52
(Intercept)	46.62				
Bayley-III CLC	0.52	0.21, 0.83	**.002**		
Age at Bayley-III	-1.26	-6.68, 4.16	.642		
Male	4.94	-1.82, 11.70	.147		
Birth weight	0.00	-0.01, 0.00	.356		
Gestational age	0.73	-1.75, 3.22	.555		
IMD (upper:lower band)	1.97	-5.19, 9.14	.581		
MRI injury score	-1.65	-3.44, 0.13	.069		
Model 3				0.36	377.04
(Intercept)	66.83				
Bayley-III CLC	0.49	0.18, 0.79	**.002**		
Age at Bayley-III	-1.44	-6.74, 3.87	.587		
Male	3.38	-3.47, 10.24	.325		
Birth weight	0.00	-0.01, 0.00	.318		
Gestational age	0.40	-2.07, 2.86	.747		
IMD (upper:lower band)	2.41	-4.61, 9.43	.492		
MRI injury score	-0.43	-2.69, 1.84	.706		
NE grade (severe:moderate)	-8.46	-18.43, 1.51	.094		

The regression coefficients were adjusted for: (1) sociodemographic data [Model 1]; (2) variables in Model 1 and neonatal brain MRI injury score [Model 2]; and (3) variables in Model 2 and NE grade [Model 3].

Note. NE = neonatal encephalopathy secondary to perinatal asphyxia; IMD = Index of multiple deprivation.
